# Kinase inhibitors: look beyond the label on the bottle

**DOI:** 10.20517/cdr.2019.80

**Published:** 2019-12-19

**Authors:** Paul Dent, Andrew Poklepovic, Laurence Booth, John F. Hancock

**Affiliations:** ^1^Department of Biochemistry and Molecular Biology1, Virginia Commonwealth University, Richmond, VA 23298-0035, USA.; ^2^Department of Medicine, Virginia Commonwealth University, Richmond, VA 23298-0035, USA.; ^3^Department of Integrative Biology and Pharmacology, University of Texas Health Science Center, Houston, TX 77030, USA.

**Keywords:** Autophagy, histone deacetylase, chaperone, cancer

## Abstract

The majority of scientists working in the field of cancer experimental therapeutics recognize that many drugs that claim to be “specific” for one target enzyme in fact regulate to varying degrees the activities of other additional protein targets. Some of these targets are known and are recognized as being an essential component of a drug’s biology. However, many other targets fall into the category of “unknown unknowns”. Thus, the collective therapeutic outcome for almost all clinically relevant drugs is reliant on both the claimed primary and secondary “on” targets as well as some of the unexpected unknown “off” targets. This review discusses the biology of several FDA approved cancer therapeutic drugs whose initial reported targets only represented the tip-of-the-iceberg in terms of how each agent acted as an anti-tumor drug. The review also discusses a putative thorough pre-visualization methodology for drug-based research, prior to executing any wet work. These approaches should be performed in an agnostic fashion and be based in part on the clinically safe drug’s C max and its area under the curve in a patient. Based on tumor heterogeneity, considerations of how to approach developmental therapeutics in the age of “personalized medicine” are also discussed.

## Introduction

**Ancient History, RAF-1, SRC, and the ERK1/2 pathway:** In January 1992, Dr. Dent arrived in Charlottesville, Virginia, to begin his postdoctoral work with Dr. Thomas Sturgill. He was handed a project that had laid fallow for a year; to understand what role the kinase, then called c-RAF-1, was playing within the ERK1/2 pathway. At that time, it was believed that RAF-1 was downstream of ERK1/2^[1]^. Furthermore, based on the structure of its active site, the kinase family most similar to this serine/threonine kinase was postulated to be the non-receptor tyrosine kinase family related to c-SRC, i.e., tyrosine kinases^[2]^. Subsequently, the Sturgill lab and another group contemporaneously published that RAF-1 was the kinase that phosphorylated and activated MEK1/2^[3,4]^.

**The development of “specific” kinase inhibitors:** Even in the early 1990s, it was recognized that high levels of ERK1/2 activity in a tumor cell predicted for a cell that grew more rapidly, could invade more rapidly, and was more resistant to chemotherapeutic treatments^[5,6]^. Hence, the development of small molecule inhibitors of RAF-1, as well as B-RAF, began, as did work on developing inhibitors of MEK1/2. At that time, it was believed that the MAP kinase family enzymes, e.g., ERK1/2 and JNK1/2/3, were undruggable due to their relatively similar active sites. The first inhibitor of any MAP kinase pathway was PD098059; this agent blocked pathway signaling at the level of MEK1/2^[7,8]^. What was particularly interesting about PD098059 was that its actions were not at the active catalytic site, as would normally be expected, but that it acted to prevent RAF-1 from phosphorylating MEK1/2^[9]^. Furthermore, the chemical was shown to inhibit cyclooxygenase activity, as well as expression of these enzymes, and could inhibit MEK5 in the ERK5 “big” MAP kinase pathway^[10-12]^. Shortly after PD098059 became an established pharmacological tool, a new MEK1/2 inhibitor became available, U0126^[13,14]^. U0126 could prevent MEK1/2 phosphorylation as well as block MEK1/2 catalytic activity. Fast forward to 2019, and several MEK1/2/5 inhibitors are now FDA approved cancer therapeutics, all having a similar mechanism of action to that of U0126^[15,16]^. Because they are not pure catalytic site inhibitors, the inhibitors of MEK1/2/5 exhibit a high level of on-target efficacy.

**Sorafenib is a switch hitter:** By the mid-1990s, Bayer announced it had developed an inhibitor of RAF-1, BAY43-9006^[17-19]^. Subsequently, the compound was named sorafenib, and at approval it was marketed under the name Nexavar. Initial studies with sorafenib, before its toxicity profile was well understood in patients, often used the drug at high non-physiologic concentrations, e.g., 10 M^[20,21]^. In addition, pre-clinical studies often used sorafenib, rather than the more soluble clinically relevant sorafenib tosylate, which further complicated the literature in understanding the biology of this drug. As would have been predicted from earlier data, sorafenib was subsequently shown to inhibit class III receptor tyrosine kinases such as PDGFRs and VEGFRs^[22-25]^. Thus, based collectively on all of these findings, the biology of the drug sorafenib is clearly highly complex.

One observation made by several groups was that sorafenib could rapidly induce an endoplasmic reticulum (ER) stress response, as judged by elevated auto-phosphorylation of PKR-like endoplasmic reticulum kinase (PERK) and phosphorylation and inactivation of the translational regulator eIF2^[26,27]^. Autophagosome formation could be observed as a downstream read-out of the ER stress signal^[26-28]^. However, neither RAF family kinases nor class III receptor tyrosine kinases can be simplistically linked to the regulation of ER stress signaling and PERK activity. From our own work, we became aware that another class III receptor tyrosine kinase inhibitor, pazopanib, which does not inhibit RAF-1, also increased PERK and eIF2 phosphorylation, and enhanced autophagosome formation^[29,30]^. A partial resolution of this unexpected data came from studies using the celecoxib derivative OSU-03012 (AR12). AR12 was originally proposed to be an inhibitor of PDK1, downstream of PI3K and upstream of AKT T308^[31]^. Our studies demonstrated that the biology of PDK1 and of OSU-03012 did not overlap^[32,33]^. As OSU-03012, in a fashion similar to sorafenib and pazopanib, was also increasing both PERK and eIF2 phosphorylation, we began to explore whether this novel agent was in fact acting as an inhibitor of protein chaperones. PERK activation is prevented by the ER located chaperone GRP78 (BiP, HSPA5)^[34,35]^. When high levels of denatured proteins are present, they interact with GRP78, which then dissociates from PERK, permitting PERK to signal to eIF2 and to suppress protein translation^[34-36]^. Once GRP78 and other effectors have either restored protein conformation or have routed misfolded proteins to degradative pathways, GRP78 re-associates with PERK, turning the stress signal off. OSU-03012 was discovered to be a drug that could inactivate GRP78 function, with the GRP78 protein itself exhibiting apparent reduced expression on SDS-PAGE Western blots^[37]^. Structurally, OSU-03012, sorafenib, and pazopanib all share, at least to the eyes of a non-organic chemist, similar chemical moieties and ring structures, and it was hypothesized that sorafenib and pazopanib might also be acting as chaperone inhibitors. Several observations were then made: (1) sorafenib, pazopanib, and OSU-03012 all had sub-500 nM IC_50_ inhibitory concentrations *in vitro* against the ATPase activities of HSP90 and HSP70^[38]^; (2) when drug-treated chaperones were fixed in place and directly immuno-stained, it was discovered that, whilst the detection of epitopes in the COOH- and central portions of the chaperones remained unchanged, those at the ATP binding NH2-terminus became masked; and (3) sorafenib, pazopanib, and OSU-03012 could all dock with the HSP90 and HSP70 ATP-binding sites in silico^[38-40]^. Hence, to fully understand the biology and therapeutic actions of sorafenib now requires appreciation of the expression and activity of class III receptor tyrosine kinases, RAF kinase activities, and chaperone protein expression and function.

During this oncology-based research, it was noted that chaperone proteins also play essential roles in the life cycles of all pathogenic human viruses. As long ago as 1991, studies demonstrated that expression of GRP78 was essential for HIV replication^[41]^. In studies using sorafenib, pazopanib, and OSU-03012 in the context of being chaperone inhibitors, numerous human pathogenic viruses exhibited reduced replication *in vitro*, including drug-resistant forms of HIV, influenza, Junín, Marburg, ebola, and measles^[42,43]^. Thus, if properly formulated for additional clinical trials, OSU-03012 via chaperone inhibition could technically become a valued novel anti-viral drug.

**The thymidylate synthase inhibitor Pemetrexed also is a switch-hitter:** The drug pemetrexed, trade name Alimta, was developed to inhibit thymidylate synthase^[44]^. The drug is used as standard of care in the treatment of non-small-cell lung cancer in combination with carboplatin, and compendium listed for the treatment of ovarian cancer. In the latter part of the 2000s, one of the original researchers who developed the drug, Dr. Richard Moran, performed additional mechanistic studies which greatly expanded the possibilities for rationally utilizing pemetrexed in novel drug-combination chemotherapies^[45,46]^. Dr. Moran’s group discovered that not only was pemetrexed a thymidylate synthase inhibitor within the pyrimidine synthetic pathway, but that it also inhibited a folate-dependent enzyme in *de novo* purine synthesis, aminoimidazole carboxamide ribonucleotide formyl-transferase (AICART). An essential substrate of AICART, 5-aminoimidazole-4-carboxamide ribonucleotide (ZMP), accumulated in pemetrexed treated cells. ZMP can act as an allosteric activator of the AMP-dependent protein kinase (AMPK), a kinase which senses elevated AMP levels in response to ATP depletion. In parallel to ZMP effects, by dysregulating pyrimidine and purine biosynthesis, pemetrexed causes a DNA damage response, activating ataxia telangiectasia mutated (ATM). ATM can phosphorylate the alpha subunit of the AMPK at threonine 172, causing the latter’s catalytic activation^[47]^. Hence, through phosphorylation and allosteric mechanisms, pemetrexed induces a strong cellular “ATP-deficiency” stress response. This results in the dephosphorylation and inactivation of mammalian target of rapamycin (mTORC1), activation of ULK1, increased ATG13 S318 phosphorylation, and the formation of autophagosomes^[48]^.

An obvious postulate, if researchers were to rationally develop novel drug combinations with pemetrexed, they would manipulate cellular signaling pathways that would reinforce independently the primary actions of that drug. As noted in the above sections, one obvious candidate drug to combine with pemetrexed is sorafenib. As a single agent, sorafenib does not activate to any appreciable levels either ATM or AMPK. However, as a kinase and chaperone inhibitor, sorafenib acts to reduce signaling via the ERK1/2 and PI3K/AKT pathways that will lead to mTORC1 and p70 S6K inactivation, and, by suppressing GRP78 function, it causes eIF2 phosphorylation. Inactivation of eIF2 combined with inactivation of p70 S6K causes the levels of cytoprotective proteins with short half-lives, e.g., MCL-1 and BXL-XL, to decline, and enhances the transcription of a small subset of stress-related regulatory genes such as those that facilitate autophagosome formation, e.g., Beclin1 and ATG5^[49-51]^. Furthermore, it was through these complementary mechanisms of drug action that pemetrexed and sorafenib interacted to synergistically kill tumor cells. Neither drug, based on its initial “on” target-designation, by the pharmaceutical companies, would have been rationally combined with the other agent. The pemetrexed plus sorafenib drug combination underwent phase I evaluation with multiple solid tumor patients having prolonged partial responses or stable disease (> 6 months), and one breast cancer patient having an initial complete response (NCT01450384). Success of this first trial resulted in a new phase II trial focused on triple negative breast cancer, with multiple patients in this trial also exhibiting partial responses or extended stable disease (NCT02624700). Owing to a repositioning by Lily, the owners of pemetrexed, this trial was unfortunately prematurely closed.

**The long and winding road linking neratinib and RAS: tales of the most unexpected.** The drug neratinib (HKI-272) was developed to be a high affinity irreversible inhibitor of EGF receptor family tyrosine kinases^[52,53]^. Neratinib was shown to be an efficacious adjunct therapy for HER2+ breast cancer, was FDA approved, and trades under the name Nerlynx. Another high affinity irreversible inhibitor of EGF receptor family tyrosine kinases, afatinib, has also been approved for the treatment of non-small-cell lung cancer that expresses a mutant activated EGF receptor^[54,55]^. Although there are significant differences in the structures of afatinib and neratinib, they both irreversibly bind to ERBB1 (HER1), ERBB2 (HER2), and ERBB4 (HER4).

Our initial studies with afatinib and neratinib took place in the context of combining these drugs with the Janus kinase (JAK1/2) inhibitor ruxolitinib. Both of these irreversible EGF receptor family inhibitors synergized with ruxolitinib to kill breast cancer as well as other carcinoma cells^[56]^. Parallel contemporaneous studies were also studying the biology of afatinib in non-small-cell lung cancer cells that express a mutant activated EGF receptor, and wild type and afatinib-resistant clones of the cells were generated *in vivo*^[57]^. The initial characterization of the afatinib resistant clones, using siRNA approaches, demonstrated that enhanced signaling by ERBB3, c-MET, and c-KIT were collectively responsible for maintaining cell viability. Unlike the afatinib-resistant clones, collective knock down of ERBB3, c-MET, and c-KIT did not reduce the viability of wild type clones.

Based on our prior experience with other drug combinations whose efficacy is enhanced by pan-EGF receptor family inhibitors, we then asked whether the afatinib-resistant cells were also resistant to the first-generation EGFR/HER2 inhibitor lapatinib and to the novel irreversible inhibitor neratinib. Compared to afatinib, lapatinib exhibited little efficacy at killing wild type or resistant lung cancer cells, while neratinib exhibited a similar killing efficacy in the wild type clones to that of afatinib^[58,59]^. To our surprise, while the *in vivo* generated afatinib-resistant clones remained resistant to afatinib *in vitro*, the same cells were still sensitive to neratinib having a significantly greater mean level of killing in all of the different resistant clones. In other words, although both drugs were designed to inhibit the same EGF receptor family targets, neratinib must clearly have additional targets through which it can cause tumor cell death in the afatinib-resistant lung cancer cells. This was independently confirmed when we discovered that afatinib and neratinib could be combined to enhance tumor cell killing^[58]^.

The inclusion of control data for any scientific experiment is essential. As we began to explore the biology of neratinib in the wild type and afatinib-resistant lung cancer cells, several startling observations were made^[58]^. We already knew that the afatinib-resistant cells expressed lower levels of ERBB1 compare to the wild type cells, and then discovered that, in all clones, wild type and resistant, neratinib rapidly reduced the protein levels of EGF receptor family members. The inclusion of negative control data in any study adds rigor to data interpretation. It was therefore surprising to discover neratinib also reduced the protein expression of our negative controls c-MET and c-KIT, receptor tyrosine kinases that do not bind neratinib^[60,61]^. The mechanisms of down-regulation were not identical, with down-regulation of ERBB1 occurring rapidly and requiring a ubiquitination step, whereas down-regulation of c-MET was slower and did not require ubiquitination. For both receptors, knock down of Beclin1 or ATG5, i.e., autophagosome formation, prevented degradation.

Receptor tyrosine kinases on the cell surface are believed to cluster in quaternary structures, which aids in the transmission of extracellular signals into the cell^[62]^. On the inner leaflet of the plasma membrane are large and small GTP binding proteins that facilitate receptor signals leaving the plasma membrane environment and entering the cytoplasm. From a cancer therapeutics perspective, proteins of the RAS family and the G_q_ and G_11_ proteins can become mutated, losing their GTPase activities and thus being permanently GTP bound, and hence permanently signaling^[63,64]^. We hypothesized that, as neratinib was causing the degradation of transmembrane receptors, it may also bring along fellow-traveler membrane-associated proteins, i.e., K-/N-/H-RAS. We employed multiple approaches to test this hypothesis. First, we utilized immunofluorescent staining of cells, examining the initial localization of K-RAS, and then any changes that occurred in its localization or co-localization with autophagy regulatory proteins after neratinib exposure^[58]^. Secondly, we used transient transfection to express K-RAS V12-GFP and K-RAS V12-RFP, and then determined whether neratinib could alter their localization within a cell^[65]^. Third, using an endomembrane mCherry tagged protein and K-RAS V12-GFP, the coefficient of GFP movement from the plasma membrane to endomembrane structures was calculated^[66]^. Using previously established approaches, we validated our K-RAS and N-RAS antibodies against their advertised target proteins; knock down of either protein resulted in reduced immunofluorescence staining for K-/N-RAS but did not reduce the expression of ERK2^[67]^.

Neratinib caused endogenous K-RAS and N-RAS to localize in vesicles within the cell, and to subsequently reduce the total amount of each protein within the cell by ~30%^[58]^. This effect could be enhanced using histone deacetylase inhibitors or by inhibitors of phosphodiesterase 5 such as sildenafil (trade name, Viagra). The vesicles co-stained for phosphorylated ATG13; ATG13 phosphorylation is the gatekeeper step leading to the formation of autophagosomes. Knock down of Beclin1 or ATG5 prevented the degradation of K-RAS and N-RAS. In cells transiently transfected to express K-RAS V12-GFP and K-RAS V12-RFP, neratinib caused both fluorescent proteins to rapidly localize within intensely staining intracellular vesicles^[65]^. In the majority of cells, both the GFP and RFP tags colocalized, producing a yellow image. In fewer cells, observations indicated that, whilst the majority of vesicles co-stained yellow, individual red punctate vesicles were also visible, suggesting that some of the RAS proteins were being directly delivered to the lysosome. This resulted in additional studies that demonstrated LC3-associated phagocytosis played a key role in the biology of neratinib^[68]^. Neratinib treatment of Madin-Darby Canine Kidney (MDCK) cells expressing K-RAS V12-GFP, which localized in the plasma membrane, and with mCherry-CAXX, which localized on an endomembrane, caused a rapid mis-localization of the GFP-tagged RAS protein from the plasma membrane to the endomembrane. After a longer 24- or 48-h neratinib exposure, the fluorescence staining intensity of mCherry-CAXX remained unaltered, whereas GFP/RAS fluorescent staining was abolished, i.e., the mutant K-RAS protein had been almost completely degraded via the actions of neratinib. This finding is also a further confirmation that EGF family receptors cannot be the only target of neratinib as, when compared to carcinoma cells, MDCK cells express relatively low levels of their endogenous canine EGF receptor^[69]^.

**Neratinib and its MAP4K targets:** In 2011, Davis *et al*.^[60]^ published an extensive report in which approximately forty drugs and approximately four hundred kinases were interrogated *in vitro* and in silico to determine the relative IC_50_ of each chemical against each kinase. For afatinib, essentially, only the EGF receptor family of tyrosine kinases were targets, implying afatinib has a specificity of target almost unique amongst the tested drugs in that study. In contrast to afatinib, neratinib had more universal tastes, and was shown to inhibit not only the EGF receptor family, but also multiple serine/threonine MAP4K family members, some with similar IC_50_ values to those for the receptor tyrosine kinases. As another example, neratinib is designed to block a tyrosine kinase but also impacts serine/threonine kinases, while sorafenib is designed to block a serine/threonine kinase but also impacts tyrosine kinases. Klaeger *et al.*^[61]^ reported similar approaches examining neratinib target specificity. Enzymes shown to be inhibited by neratinib within the same concentration range as EGF receptor family tyrosine kinases include MST3, MST4, MAP4K5, and MAP4K3. The safe maximal plasma concentration of neratinib in a patient is ~150 nM and proposed *in vitro* IC_50_ inhibitory concentrations of neratinib for kinases below 100 nM also include: GCN2, MAP4K1, MAP3K4, MST2, and YSK4. Thus, understanding the true biology of neratinib, through a multitude of effectors, has now become considerably more challenging.

For the kinases neratinib is claimed to inhibit at sub-10 nM concentrations, the following is known: MST3/4 control the apical brush border of epithelial cells. The major dose limiting toxicity of neratinib is diarrhea, arguing that neratinib is acting in an on-target MST3/4-dependent fashion to cause this event. MST3/4 also coordinate the phosphorylation of cytoskeletal proteins such as Ezrin/Radixin/Moesin family to regulate plasma membrane ruffling^[70,71]^. MAP4K5 is an apical kinase that interacts with GTP binding proteins downstream of G protein coupled receptors (GPCR), and links GPCR signaling into MAPK pathways. MAP4K5 and MAP4K3 phosphorylate and activate the LATS1/2 kinases that in turn phosphorylate and inhibit YAP/TAZ, the main co-transcription factor effectors of the Hippo pathway^[72,73]^. MAP4K3 has also been shown to play an important role in amino acid signaling to mTOR/p70 S6K, which in turn will regulate autophagosome formation^[74]^.

As observed in our prior work, knock down of Beclin1 or of an additional autophagosome regulatory protein, ATG16L1, reduced the ability of neratinib to reduce ERBB1 and K-RAS expression^[68]^. MAP4K enzymes are expressed in both solid and liquid tumor cells. In carcinoma cells that express EGF receptor family proteins, and in hematopoietic cancer cells lacking ERBB1/2/4, neratinib reduced K-RAS expression as well as the phosphorylation of MST1/2/3/4 and Ezrin by ~30%. Neratinib increased the phosphorylation of the downstream targets of the MST kinases, LATS1/2, as well as of the downstream targets of LATS1/2, the co-transcription factors YAP and TAZ, also by ~30%. Despite only a measured ~30% enhancement of YAP S127 and YAP S397 phosphorylation, neratinib caused the majority of YAP protein to translocate from the nucleus and into the cytosol, where it co-localized with Beclin1, i.e., autophagosomes, and YAP/TAZ protein levels were reduced. Thus, unexpectedly from the original designation of neratinib being only an inhibitor of the EGF receptor family, this drug is also capable of downregulating mutant RAS proteins and reducing the co-transcription factor functions of YAP and TAZ in the Hippo pathway.

**Triple-drug combinations based on novel mechanisms of drug action:** Thus far, in this review, we discuss drugs individually as well as when rationally combined with a second agent. As can be expected with any drug or drug combination, the development of drug resistance by a genetically unstable tumor cell will ultimately limit the efficacy of the primary drug/drug combination. Thus, the rational inclusion of a third drug, either to circumvent a primary mode of resistance evolution or simply to further enhance the initial lethality of a two-drug combination, is a logical approach.

Two of the drug combinations Dr. Poklepovic has translated from the Dent laboratory to the bedside are pemetrexed plus sorafenib, and the multi-kinase inhibitor regorafenib plus the PDE5 inhibitor sildenafil. Pre-clinical studies examining the biology of pemetrexed plus sorafenib treated tumors revealed that the surviving cells had activated their EGF receptors^[75,76]^. Molecular knock down of at least two EGF receptor family members was required to enhance pemetrexed plus sorafenib lethality *in vitro*, and the pan-receptor inhibitors lapatinib, afatinib, and neratinib all significantly enhanced two-drug lethality^[75]^. Of note, the third-generation specific inhibitor of mutant EGF receptor signaling osimertinib (trade name Tagrisso) was considerably less effective at promoting two-drug killing^[75]^. Pre-clinical studies with regorafenib plus sildenafil in gastrointestinal tumor cells expressing mutant K-RAS proteins revealed that the surviving cells had activated not only their EGF receptors but also PDGFRs, c-MET, and c-KIT^[65]^. Clearly, to improve the efficacy of regorafenib plus sildenafil, we needed a drug which could “attack” multiple receptor tyrosine kinases and, ideally, also suppress the actions of mutant K-RAS. We determined, not surprisingly, that neratinib *in vitro* and *in vivo* could significantly enhance the anti-tumor efficacy of regorafenib plus sildenafil. The phase I trial combining regorafenib plus sildenafil has recently closed, with a number of patients receiving significant life-extending benefits (NCT02466802). It is hoped that additional studies, with this combination including neratinib, can be performed in the future to determine safety and efficacy.

**Pre-visualization of experimental therapeutics studies:** A pre-visualization approach is required prior to the use of any therapeutic agent or combination of agents for mechanistic assessments to ensure, as much as possible, that the drug concentrations being used *in vitro* are within “the clinically relevant safe concentration range in a patient’s plasma” Initially, pharmaceutical companies with libraries of compounds will perform *in vitro* and in silico kinase profiling to determine putative targets. In addition, RNA-seq/mass spectrometry approaches can be utilized to define other potential non-kinase targets.

For drugs that have not yet entered the clinic, at the very least, prior to any wet work, the LD_10_ and assessments of normal tissue toxicities of the drug should be determined in rodents to provide a “guesstimate” foundation for performing mechanistic *in vitro* pre-clinical studies. The use of clinically unachievable drug concentrations for *in vitro* studies, from the standpoint of precise targeting, is detrimental to the successful translation of any agent. As pointed out for neratinib, some of its serine/threonine kinase targets have IC_50_ values in the very low nanomolar range, and some in the intermediate 10-100 nM range. However, there are additional neratinib targets in the 100-500 nM range and above, and whose inhibition would potentially confound a true definitive determination of the drug’s mechanisms of action. Hence, our initial research with neratinib, “searching for any effects”, started using *in vitro* concentrations of 500 nM (too high), which we then reduced to 100 nM (highest for any translational relevance) and 50 nM (ideal). It is worth noting, however, that identical effects on EGF receptor and K-RAS downregulation were observed at all neratinib concentrations tested, although the amplitude of the observed effects was reduced at lower concentrations of the drug.

Pharmacologists who are a component part of any drug-development team, during the initial phase I trial of any agent, will measure the plasma concentration of the drug over time at each dose level. The maximum peak plasma concentration of the drug is termed the C max. Pharmacologists also measure the time it takes to reach the C max, and the elimination half-life of the drug, collectively providing a sigmoidal plot, the area under the curve (AUC). For example, a drug has the potential to generate a C max in the micromolar range whilst in parallel also having a very rapid elimination/metabolism into the low nanomolar range, e.g., sildenafil. All drugs, to a greater or lesser extent, also bind to plasma proteins; these assays are usually performed prior to clinical studies and provide evidence to suggest the amount of “free” drug in the plasma that could go on and effect biological outcomes. Thus, before any new *in vitro* wet work is performed, pre-visualization planning requires a knowledge of the safe plasma C max for the drugs to be combined, which is essential if the subsequent *in vitro* studies are to have any putative translational relevance. Furthermore, if the AUC indicates a rapid elimination of the drug, drug concentrations significantly below the C max need to be employed. Furthermore, because drug combinations in phase I trials do not initially dose the two agents at each of their maximum safe tolerable doses, this also needs to be considered when designing new *in vitro* experiments and interpreting the relative importance of any data collected.

The amount of free versus protein-bound drug must also be considered, albeit with certain caveats. For example, the safe plasma C max of sorafenib, depending on the data from various trials, is approximately 13 micromolar for a single 400 mg standard of care dose^[77,78]^. It should be noted that many liver and kidney cancer patients do not tolerate the 400 mg twice daily dosing of sorafenib and take a dose-modified 200 mg twice daily or even 200 mg daily, yet these patients can still routinely present with measurable anti-tumor effects. *In vitro* binding of sorafenib to plasma proteins indicates that 99.5% of the sorafenib is “protein bound”. If we assume a 200 mg dose of sorafenib with a C max of approximately 6.5 µM generates a clinical effect, this would imply a plasma free-drug C max of approximately 30 nM. However, *in vitro* exposure of liver and kidney cancer cells to 30 nM sorafenib tosylate does not appreciably alter liver or renal carcinoma cell biology^[79]^. *In vitro* studies culturing tumor cells in 100% human serum demonstrated that a 2 μM concentration of sorafenib tosylate had single agent as well as combinatorial anti-tumor efficacy with HDAC inhibitors^[79]^. A similar observation for the incongruence between drug action, plasma C max, protein binding and anti-neoplastic activity has been made for the Brunton’s Tyrosine Kinase inhibitor ibrutinib^[80]^. Thus, whether “plasma protein binding” can be used as a precise measure to assess the levels of biologically active “free” drug, as compared to the C max and AUC, needs further study.

## Conclusions

All drugs have primary targets, and many have known secondary targets that collectively can explain many of the biological actions of the agent. The issue for the rational use of drugs as single agents, or in rational combinations with other modalities, is that, during drug development, many researchers and drug companies ignore the “unknown unknowns” that are implicit in the medical development of all small molecule compounds. Information in this review therefore suggests drug development needs to simultaneously progress along two parallel tracks. One track, straightforward, is to develop an already identified compound for its “specific” target of interest. However, a second track, less straightforward and requiring an open mind, is to agnostically interrogate the same compound using cells that express very little or none of the “specific” target enzyme. For example, that neratinib can alter tumor cell biology in blood cancer cells which express almost no members of the EGF receptor family argues precisely for this concept. In parallel, gene editing strategies such as CRISPR/Cas9 could also be utilized to remove the “primary” target kinase from a cell and hence permit comparison isogenic cells that express the kinase of interest. Using these genetically modified cells for drug-testing either *in vivo* as tumors or as *in vitro* 3D co-culture model systems then provides a further level of mechanistic definition for the targets of drug action, i.e., performing studies in conditions that mimic the *in vivo* tumor environment.

Over the past 20 years, many drug companies have worked to generate highly “specific” drugs for specific mutant targets, and yet many of those highly specific agents have ultimately only found clinical approval in tumor cell types which are exquisitely addicted to the signals generated by that specific mutated enzyme, a case in point being osimertinib^[75,81]^. Osimertinib is efficacious against lung cancer cells expressing a mutated EGF receptor but is inactive in squamous head and neck tumor cells that are addicted to high levels of signaling by a wild type EGF receptor^[82]^.

In the phase I trial combining pemetrexed and sorafenib, the second patient recruited, in the lowest dose cohort, exhibited a profound anti-tumor response in all of her cutaneous metastatic lesions. This intervention represented the tenth modality she had received for her triple negative mammary carcinoma. After five months of treatment, one small portion of one of her tumors progressed. What became obvious at this time, however, was that, of the original cutaneous tumors, over 90% of the tumor mass had been eliminated^[75]^. Most of the lesions exhibited a complete response and had not regrown in the five months on therapy. Several small lesions neither grew nor were reduced by the therapy. Only one small portion of one lesion regrew. Thus, this patient had at least three separate clonal variants of her mammary tumor. In the present day, “personalized medicine” is used in both professional and non-professional publications with the stated goal of being able to precisely tailor the therapeutic drug regimen to the biology of each patient’s tumor. However, for the patient under discussion, or in fact for many other tumor types that initially present with multiple clonal variants, e.g., glioblastoma multiforme, the concept of personalized medicine is likely to be redundant. For this particular patient, a personalized medicine approach would, hypothetically, mean choosing between which of the three clonal variants of her tumor she most wanted to be treated. Thus, if we are to successfully control “cancer” for the vast majority of patients in the real world, whose tumors are not exquisitely addicted to a single mutated driver protein, we must rationally employ agents alone or in combination that have broader specificities, so that the vast majority of the clonal variants of each specific patient’s tumor can be simultaneously attacked and killed, thereby prolonging progression free survival and overall survival.

## Declarations

### Copyright

© The Author(s) 2019.
